# Tranexamic Acid Subcutaneously Administered with Epinephrine and Lidocaine in Upper Blepharoplasty: A Randomized Double-Blind Control Trial

**DOI:** 10.1007/s00266-024-04112-z

**Published:** 2024-05-24

**Authors:** Thitinan Chaichumporn, Puritat Kanokkangsadal, Achariya Sarovath

**Affiliations:** 1 Division of Plastic and Maxillofacial, Department of Surgery, Faculty of Medicine, Ramathibodi Hospital, Mahidol University, 270 Thanon Rama VI, Thung Phaya Thai, Ratchathewi, Bangkok, 10400 Thailand; 2 Center of Excellence in Applied Thai Traditional Medicine Research, 99/209, Faculty of Medicine, Thammasat University, Klongnueng, Klongluang, Pathumtani 12120 Thailand

**Keywords:** Epinephrine, Subcutaneous injection, Tranexamic acid, Upper blepharoplasty

## Abstract

**Background:**

Eyelid surgery is one of the top five aesthetic procedures. It is performed to improve both appearance and function, but intraoperative bleeding leads to adverse events which perturb patients. The objective of this study was to demonstrate the efficacy of TXA combined with epinephrine in decreasing intraoperative blood loss and postoperative inflammation.

**Methods:**

This prospective randomized control trial was performed on the 30 eyelids of 15 patients who underwent upper blepharoplasty. One of each patient’s eyes was randomly assigned to the TXA group, and the other eye was in the control group. Eyes in the TXA group were given 2% lidocaine with epinephrine (1:100000) mixed with TXA (50 mg/ml) in 1:1 mixture subcutaneously as a local anesthetic. The eyes in the control group received 2% lidocaine with epinephrine (1:100000) diluted with normal saline in 1:1 mixture. Intraoperative blood loss and postoperative swelling were compared between the two groups.

**Results:**

Intraoperative blood loss was significantly higher in the TXA group [4.86 (1.83) ml] than it was in the control group [2.53 (1.49) ml] (*p *< 0.001). There was no statistically significant difference between the two groups in operative time (*p *= 0.645), pain score (*p *= 0.498), lid crease (*p *= 0.548), or MRD1 (*p *= 0.626). On postoperative day 7, there was no difference in lid crease (*p *= 0.879), MRD1 (*p *= 0.463), pain score (*p *= 0.934), or ecchymosis (*p *= 0.976) between two groups.

**Conclusions:**

TXA in lidocaine with epinephrine was found to increase intraoperative bleeding compared to lidocaine with epinephrine alone, but there was no difference in postoperative swelling or ecchymosis. TXA combined with lidocaine and epinephrine injected subcutaneously should be avoided until additional relevant data are obtained. Further drug interaction study is needed.

**Level of Evidence II:**

This journal requires that authors assign a level of evidence to each article. For a full description of these Evidence-Based Medicine ratings, please refer to the Table of Contents or the online Instructions to Authors www.springer.com/00266.

## Introduction

Upper blepharoplasty is performed to treat dermatochalasis and ptosis and in Asian people, for aesthetics [1]. Eyelid surgery is one of the top five aesthetic procedures. Most blepharoplasty operations are performed in an outpatient setting under local anesthesia. But, following the operation, many patients are concerned about swelling and ecchymosis from inflammatory response. Intraoperative bleeding affects postoperative swelling and ecchymosis [2].

The most common anesthetic drug used in upper blepharoplasty is lidocaine with epinephrine. Epinephrine stimulates alpha1 receptors, which constricts peripheral blood vessels and decreases intraoperative bleeding.

Tranexamic acid (TXA) is a lysine analog antifibrinolytic agent. It inhibits fibrin degradation and increases clot stabilization, and it may also attenuate inflammatory response [3]. In previous studies, it reduced inflammatory cytokines and biochemical markers such as IL-6, fibrin separation products, and creatine-kinase [4, 5]. TXA has been used intravenously in cardiothoracic surgery to decrease intraoperative bleeding and minimize intraoperative and postoperative blood transfusion [6]. It has been injected intra-articularly in knee surgeries to reduce bleeding [7]. Topical TXA is used in many operations to reduce blood loss and for moistening and irrigation [8, 9]. In epistaxis patients, local nasal packing soaked in TXA can reduce rebleeding comparably to anterior nasal packing [10]. TXA combined with epinephrine has been used in local wound soaking as anesthetic and to reduce bleeding [11]. TXA has been added to anesthetic drugs for subcutaneous administration in many operations, but the data on its efficacy are limited. The purpose of this study was to demonstrate the efficacy of TXA combined with epinephrine in decreasing intraoperative blood loss and postoperative inflammation.

## Patients and Methods

This study was a single center, double blinded, prospective randomized control trial in patients who underwent upper blepharoplasty by a single surgeon at Ramathibodi Hospital, between January 2020 and December 2021. The primary outcome of this study was intraoperative blood loss, and secondary outcomes were operation time, degree of postoperative inflammation assessed via swelling, ecchymosis, and pain on day 7 postoperation. This study was approved by the Human Research Ethics Committee, Faculty of Medicine Ramathibodi Hospital, Mahidol University, and was TCTR registered (TCTR20200319009).

**Fig. 1:**
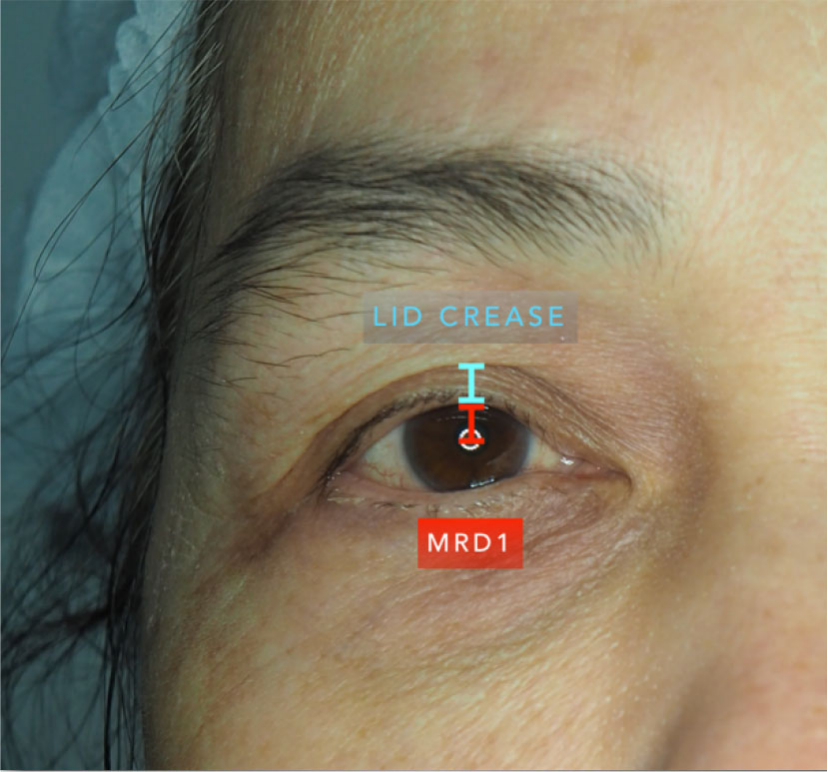
Lid crease and MRD1 evaluation

Inclusion criteria were patients who underwent upper blepharoplasty, were over 18years old, and provided written informed consent to participate in the study. Exclusion criteria were patients who had ptosis, hypertension (systolic blood pressure [SBP] > 160 mmHg), previous eyelid surgery, history of thromboembolism, seizure, TXA allergy, or were pregnant. Preoperative lid creases (mm) and marginal reflex distance 1 [MRD1] (mm) were measured with a standard caliper (Fig. 1). Photographs were taken of anteroposterior view of full face and standard view of eyes with both eyes open and gently closed.

Patients who were receiving anticoagulants were advised to discontinue taking them at least 7 days before the operation. Hypertensive patients were advised to continue their anti-hypertensive drugs and to keep their SBP under 160 mmHg.

Each patient’s eyes were randomly assigned to either of two groups, but not both to the same group, by a computer program. The first group (TXA group) was given 2% lidocaine with epinephrine (1:100000) mixed with TXA (50 mg/ml) in 1:1 mixture subcutaneously as local anesthetic, and the second group (control group) received 2% lidocaine with epinephrine (1:100000) diluted with normal saline in 1:1 mixture. The anesthetic regimen for each eye was blinded for the surgeon, the researcher, and the patients.

### Intraoperative Evaluation

**Fig. 2:**
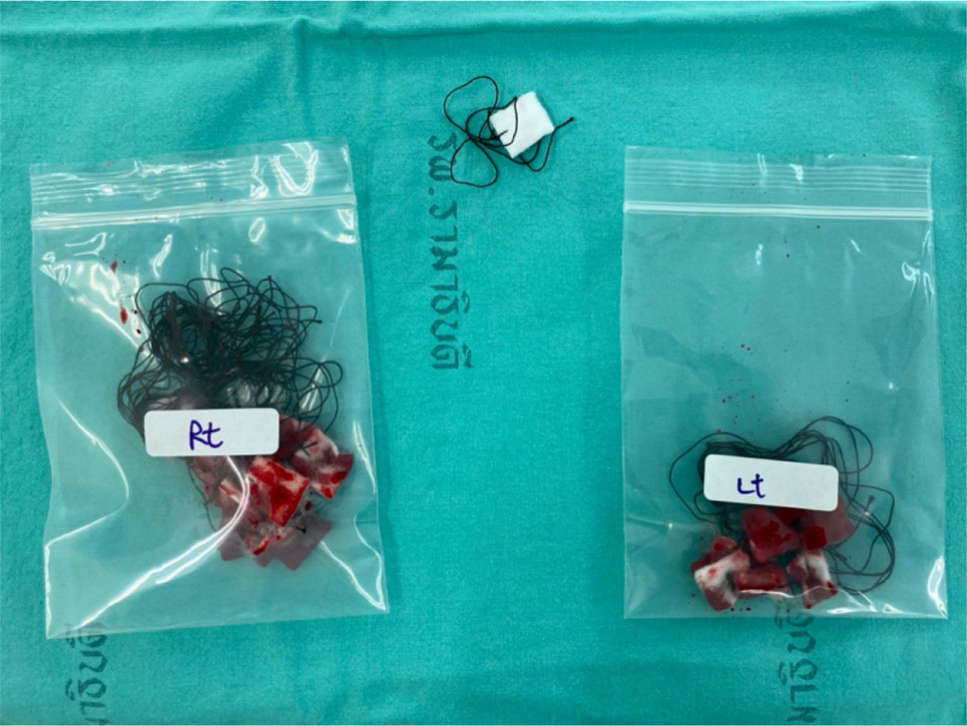
Blood-soaked cottonoids were separated for each side in zip lock bags and weighed

Upper blepharoplasty was performed under a randomized anesthetic regimen. The anesthetic medication was prepared for each eye by an unblinded scrub nurse. The operation always started on the right eye. After the eyelid was prepared using a sterile technique, the assigned anesthetic regimen was injected subcutaneously, and the surgeon waited for 10 min before starting the operation. Intraoperative bleeding was stopped with a standard electrocautery device. Intraoperative blood loss was collected with cottonoids and separated for each eye in zip lock bags to avoid evaporation (Fig. 2). After the operation, the bags with blood-soaked cottonoids were weighed by a blinded researcher, and the net total blood loss in each eye was calculated. Operation time and the amount of anesthetic drug (ml) used for each eye was recorded. Pain during anesthetic drug injection was evaluated by the patients with a visual analog scale from 0 to 10.

### Immediate Postoperative Evaluation and Management

With the patient in an upright position, anteroposterior views of full face and standard views of eyes with both eyes open and gently closed were photographed. Lid crease (mm) and MRD1 (mm) were measured. Patients were advised regarding local wound care and cold packing. Prophylaxis antibiotics and paracetamol for pain control were provided.

### 7-day Postoperative Evaluation

**Fig. 3:**
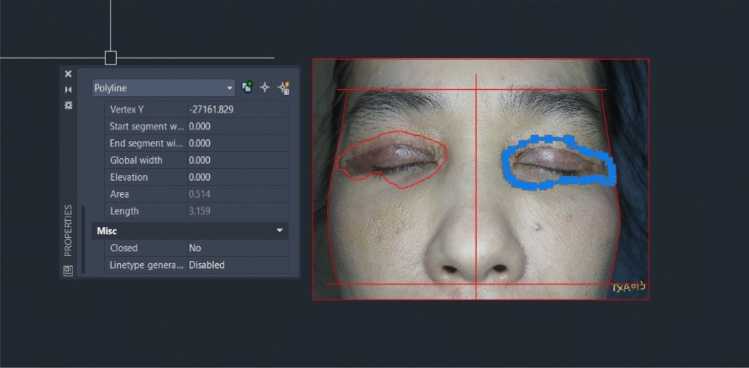
AutoCAD program calculated area of ecchymosis

On day 7 postoperation, sutures were removed, and patients were advised regarding scar prevention. Lid crease (mm) and MRD1 (mm) were re-evaluated. With the patient in an upright position, anteroposterior views of full face and standard views of eyes with both eyes open and gently closed were rephotographed. Pain score was evaluated. Ecchymosis was independently reviewed and evaluated from the photographs by the researcher, a plastic surgery resident, and an experienced plastic surgeon who did not perform the surgery. The area of ecchymosis was calculated in percentage compared to the periorbital area on each eye using AutoCAD (Version R.47.M.87, Autodesk, USA) (Fig. 3).

### Statistical Analysis

Descriptive statistics were analyzed using mean, percentage, and SD. Independent pair *t* test was performed to compare between the two groups, with *P* value < 0.05 indicating significant difference. Statistical analysis was carried out with IBM SPSS Statistics (Version 16.0, USA).

## Results

Eighteen patients were enrolled in this study. Three patients were excluded due to ptosis. Finally, fifteen patients with thirty eyelids were included in the study. The patient’s mean (SD) age was 57.6 (6.7) years. Fourteen patients (93%) were female. Preoperative diagnosis was dermatochalasis for all fifteen patients (Table 1).

**Table 1:** Demographics and characteristics

Data	Patients (*n*)
*Age (y)*	
30–45	1
46–60	8
> 60	6
*Sex*	
Female	14
Male	1
*Diagnosis*	
Dermatochalasis	15
Underlying disease of hypertension^a^	5
Anticoagulant usage^b^	1

^a^All hypertensive patients keep SBP < 160 mmHg.

^b^All patients discontinued anticoagulant at least 7 days before surgery.

### Intraoperative Results

The results are summarized in Table 2. Intraoperative blood loss was significantly higher in the TXA group [4.86 (1.83) ml] compared with the control group [2.53 (1.49) ml] (*p *< 0.001). There were no statistically significant differences in volume of anesthetic drug (*p *= 0.962), operative time (*p *= 0.645), pain score (*p *= 0.498), lid crease (*p *= 0.548) or MRD1 (*p *= 0.626) between the two groups, but the TXA group tended to have longer operation time [13.17 (7.86) vs. 11.94 (6.61) min] and higher pain score [4.20 (2.40) vs. 3.57 (2.65)] than the control group.

**Table 2:** Comparison between the TXA group and the control group of intraoperative and postoperative results

Parameter	TXA group (*n* = 15)	Control group (*n* = 15)	*P* value^a^
*Intraoperative*			
Volume of anesthetic drug	2.45 (0.378)	2.45 (0.383)	0.962
Blood loss (ml)	4.86 (1.83)	2.53 (1.49)	0.001
Operative time (min)	13.17 (7.86)	11.94 (6.61)	0.645
Pain score	4.20 (2.40)	3.57 (2.65)	0.498
Lid crease (mm)	6.50 (1.70)	6.15 (1.45)	0.548
MRD1	1.81 (0.85)	1.97 (0.86)	0.626
*Postoperative*			
Lid crease (mm)	6.10 (1.71)	6.20 (1.85)	0.879
Ecchymosis (%)	23.41 (9.91)	23.52 (11.19)	0.976
MRD1	2.97 (0.77)	2.77 (0.70)	0.463
Pain score	1.33 (2.19)	1.40 (2.17)	0.934

Data represented as mean (SD), ^a^Statistical analysis = Independent *t* test

### Postoperative Results

**Fig. 4:**
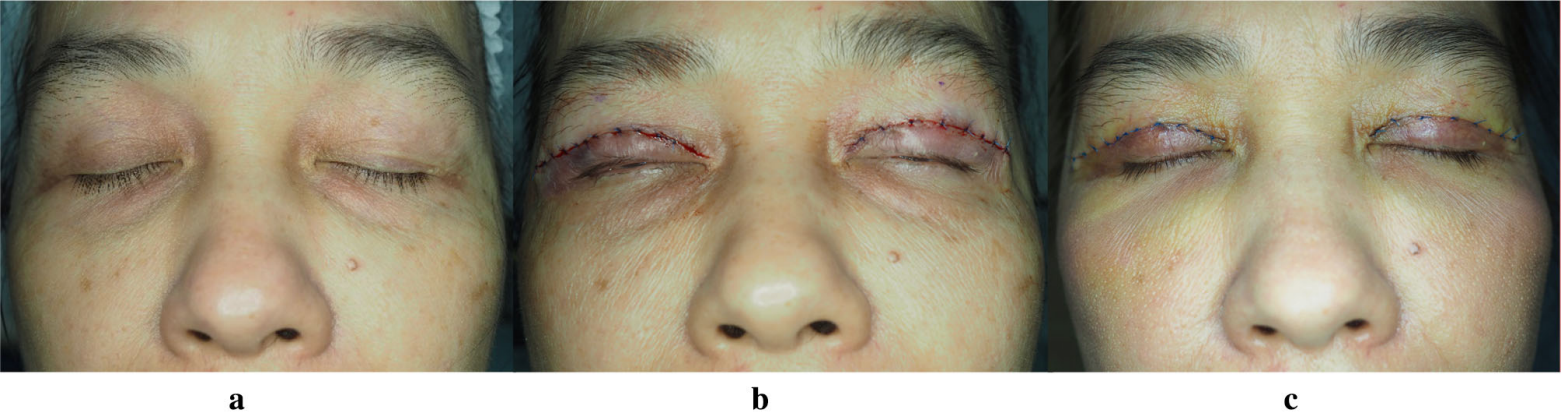
Patient with TXA on right side operation. **a** Preoperation, **b** Postoperation day0, **c** Postoperation day7

**Fig. 5:**
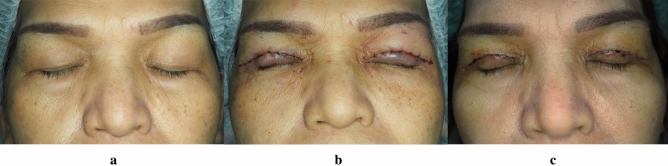
Patient with TXA on left side operation. **a** Preoperation, **b** Postoperation day0, **c** Postoperation day7

Postoperative evaluation was performed on postoperation day 7 (Figs. 4 and 5). There was no significant difference between the two groups in lid crease (*p *= 0.879), MRD1 (*p *= 0.463), pain score (*p *= 0.934), or ecchymosis (calculated as percentage of area, *p *=   0.976) (Table 2). No patients presented with postoperative complication.

## Discussion

TXA is a lysine analog antifibrinolytic agent that inhibits fibrin degradation and increases clot stabilization. Previous studies found that tranexamic acid also decreased inflammatory response. TXA is widely used in many surgical procedures to decrease blood loss during an operation.

A prospective study by Zilinsky et al. [12] showed that subcutaneous injection of TXA reduced bleeding during dermatologic surgery. In contrast, a prospective study by Sagiv et al. [13] showed that subcutaneous TXA reduced intraoperative and postoperative hemorrhaging in upper blepharoplasty only as well as a placebo. Most studies have compared between TXA + epinephrine and TXA alone [14, 15]. There is not much research comparing TXA + epinephrine to TXA +  epinephrine + lidocaine in subcutaneous anesthetic.

This prospective randomized control trial is a study of the efficacy of TXA combined with epinephrine to reduce intraoperative bleeding and postoperative inflammation when injected subcutaneously. The efficacy of TXA compared to the control was studied in the same individuals to minimize intrapersonal confounders. Interestingly, intraoperative bleeding was significantly higher in the TXA group compared to the control group. That result contradicted our hypothesis and prior study results. Perhaps, when injected subcutaneously, the acidity of TXA reduced the activity of epinephrine, or TXA molecules might have bound with epinephrine molecules or vascular adrenergic receptors, inhibiting epinephrine activity. Additional research on interaction between TXA and epinephrine is needed. Furthermore, this study did not demonstrate any difference in postoperative swelling and ecchymosis, implying zero efficacy in reducing postoperative inflammation.

A limitation of this study is the small sample size, which was due to the COVID-19 pandemic limiting the number of patients with aesthetic procedures during the study period. Further prospective studies with larger populations should be performed before drawing any conclusions.

## Conclusions

TXA in lidocaine with epinephrine was found to increase intraoperative bleeding compared to lidocaine with epinephrine alone, while there was no difference in postoperative swelling and ecchymosis. TXA combined with lidocaine and epinephrine injected subcutaneously should be avoided until additional relevant data are obtained. Further studies on drug interaction between TXA and epinephrine and larger clinical trials are needed.
